# Prospective study of early and late outcomes of extremely low birthweight in Central Saudi Arabia

**DOI:** 10.1186/s12887-018-1248-y

**Published:** 2018-08-22

**Authors:** Mostafa A. Abolfotouh, Saif Al Saif, Waleed A. Altwaijri, Mohammed A. Al Rowaily

**Affiliations:** 1 Research Training and Development Section, King Abdullah International Medical Research Center, King Saud bin-Abdulaziz University for Health Sciences - Ministry of National Guard-Health Affairs, Riyadh, Saudi Arabia; 2 King Abdulaziz Medical City, King Saud bin-Abdulaziz University for Health Sciences, Ministry of National Guard-Health Affairs, Riyadh, Saudi Arabia

## Abstract

**Background:**

Survival of preterm neonates has steadily improved over the past five decades, due to changes in the neonatal intensive care. However, in Saudi Arabia, there are no written guidelines on the definition of the lower limit of viability, and there has been a call for such a limit. The aims of this study were: (1) to determine lower limits of viability and survival in extremely low birthweight (ELBW) infants, and (2) to determine incidence of neurodevelopmental and cognitive abnormalities within 3–6 years after birth.

**Methods:**

Prospective study of all live inborn ELBW infants admitted to the neonatal unit of King Abdulaziz Medical City, Riyadh, Saudi Arabia, within 3 years [between January 1st, 2005 and December 31st, 2007] was conducted (*n* = 117). Data were collected on demographic and birth data, neonatal complications & interventions and death on discharge. Prospective follow up of all survivors was done, within 6 years after birth, to assess the outcome in terms of neurodevelopmental and cognitive abnormalities. Predictors of survival were determined using logistic regression model. Significance was considered at *p*-value ≤0.05.

**Results:**

Of all ELBW infants, 41% died before discharge. Survival rate was directly correlated with gestational age (GA) and birthweight (*p* < 0.05). The 50% limits of viability were those at 25 weeks’ gestation or with > 600 g. After adjusting for possible confounders, significant predictors of survival were birthweight (*p* = 0.001) and Apgar score (*p* < 0.001). The following impairments were reported during follow up of survivors: developmental delay (39.2%), cerebral palsy (36.2%), speech problems (33.3%), wasting (12.5%), intellectual disability (10%), visual problems (6.6%) and hyperactivity (5.6%).

**Conclusion:**

More than one-third of ELBW died before discharge from NICU, and two-thirds of survivors had one or more neurodevelopmental and/or cognitive abnormalities during their first 6 years of life. The 50% limits of viability of ELBW infants were those at week 25 of gestation or with a birthweight of more than 600 g. Birthweight could be considered as more valid than gestational age in the prediction of viability of ELBW infants. The process of care of ELBW infants in Saudi Arabia may need to be revisited taking these findings into consideration.

## Background

Over the past five decades, mortality of preterm neonates has steadily decreased [1–4]. The 50% limits of viability of infants has decreased to 23 to 24 weeks during this decade as compared to 30 to 31 weeks in the 1960s [4] This improvement in survival might be due to the changes in the methodologies of neonatal intensive care, new modes of respiratory support for neonates with respiratory distress, and development of a safety culture with the training of clinicians to work together in this culture [5].

Very preterm neonates showed significant improvement in the neurodevelopmental outcome over this period, yet this improvement lagged behind that of survival [1, 2, 4–6]. Cognitive and neurologic impairments were common among extremely preterm children at school age, as compared with their classroom peers [7, 8]. Long-term health and educational needs were required at the age of 8 years, for ELBW who were born in the 1990s [9]. Those ELBW infants, when examined for cognitive and behavioral outcomes at school age, showed an IQ that was nearly two-thirds SD below that of healthy controls [10].

Very preterm infants are influenced by multiple factors that affect their survival and long-term neurodevelopmental outcome. Those who are born at large perinatal–neonatal centers, have survival advantage, because of the availability of health care providers who have vast experience in providing perinatal and neonatal care as well as comprehensive multidisciplinary operational structure [4, 11].

The lower limit of viability was set as 22 weeks of gestational age (GA) or 500 g birth weight, by the WHO [12], 22 completed weeks (154 days) gestation and ending 7 complete days after birth, by the International Classification of Diseases [13], 22 completed weeks of gestation, (instead of 24 weeks), by the Eugenic Protection Act in Japan [14] and 23 weeks GA and/or 400 g in birthweight, by the American Academy of Pediatrics [15]. These amendments may encourage physicians to fully resuscitate these infants at the delivery room without considering the high mortality and morbidity [16].

In Saudi Arabia, there are no written guidelines on the definition of the lower limit of viability, and there has been a call for such limit [16]. Obstetric measures for the prevention of preterm delivery need to be optimized in order to decrease the morbidity and mortality associated with extremely low birthweight infants. To achieve this goal, the outcome of extremely low birth weight Saudi infants should be understood. This study aimed to determine the survival rate of extremely low birthweight infants delivered at King Abdulaziz Medical City (KAMC), at different values of birth weight and gestational age, to determine the lower limit of viability and survival outcome, and to determine the incidence of neurodevelopmental impairments and cognitive abnormalities 3 to 6 years after birth.

## Methods

### Study setting

The Neonatology Section with 40 ventilated NICU beds is considered the largest section in the Pediatric Department at KAMC. It provides a high standard of care for high risk newborn infants free of charge. Neonatal services consist of the care in NICU, and can accommodate up to 40 beds (LEVEL III) and 30 beds in ICN (LEVEL II). Admission Nursery can accommodate up to 50 beds, all operated with full capacity. The NICU accepts 23 weeks gestation to term infants requiring level II and III specialized care. The Neonatal Team consists of six consultants, two assistant consultants, ten staff physicians, two Perinatal-Neonatal fellows, and three rotating pediatrics residents. A total of 140 NICU well trained and skilful nurses are available, with a nurse to patient ratio of 1:1–2. It provides a family-centered approach to care; encompassing the parents and the sick infant as a single unit.

### Study subjects

All extremely low birthweight neonates (< 1000 g) who were admitted to the neonatal unit of the KAMC, within 3 years between January 1st, 2005 and December 31st, 2007, constituted the target of this study (*n* = 117). These children were identified from the obstetric and delivery logbook of the maternity unit. Patients’ charts were retrieved and data on maternal and neonatal demographics, clinical course and outcome (status at discharge from the unit) was extracted.

All these children were contacted to follow them up yearly till the age of six. A research coordinator was allocated for the follow up of cases via home visits in case of drop out. Every child was followed up twice by both a developmental pediatrician and a psychologist, in prescheduled visits, within the age of 3 to 6 years.

### Study design

This is a retrospective/prospective cohort study. This approach is useful for exposures that have both short term and long term outcomes. It includes exposure baseline in the past (extremely low birth weight), and a follow up period (past to present to future). Data collection went in both directions. We looked through records for birth event and started to follow up these infants into the future for neurological, cognitive and educational outcomes at different ages till the age of six.

### Data collection and variables

#### Retrospective phase

A data collection sheet was used to collect the following data:

1. Maternal reasons for the prematurity; preeclampsia, intrauterine growth restriction, preterm labor, premature rupture of membranes, or multi-fetal pregnancy (multiple gestation), previous pregnancy losses, previous preterm delivery, antepartum hemorrhage and anemia.
2. Neonatal interventions, such as; the use of antenatal corticosteroids for women at risk for preterm delivery, surfactant for the prevention and treatment of neonatal respiratory distress syndrome, postnatal steroids for chronic lung disease (CLD), and modes of respiratory support for neonates with respiratory distress (conventional ventilation, high frequency ventilation, any assisted ventilation, nasal CAPA), and red cell transfusions [5].
3. Demographic and birth data: Gestational age (< 27 weeks vs > 27 weeks), based on the date of the last menstrual period and confirmed with ultra-sonographic findings, gender, multiple birth, weight (between 450 and 600 g and 801-995 g), presentation (vertex or non-vertex), 5 min Apgar, and mode of delivery (SVD vs LSCS)
4. Outcome in terms of: death at discharge and neonatal morbidity including pneumothorax; respiratory distress, defined as the need for oxygen therapy; chronic lung disease, defined as an oxygen dependency at 36 weeks; patent ductus arteriosus, confirmed with echocardiography; episodes of sepsis, defined as clinical signs of infection with a positive blood culture; necrotizing enterocolitis (NEC); intraventricular hemorrhage (IVH) and peri-ventricular leukomalacia (PVL).

#### Prospective phase

Surviving children were assessed at 3 and 6 years of age, corrected for prematurity, by a developmental pediatrician and a psychologist. Neurological (neurosensory impairments and disabilities), cognitive, and educational outcomes were measured for children according to their current ages.

The pediatric assessment includes a neurological examination to determine outcomes such as cerebral palsy. Visual acuity was assessed by an optometrist. Modified Denver Developmental Screening test (DDST) for Arab Children [17] was used for assessment of developmental delay. Every child was assessed for the 4 areas of development (gross motor, fine motor/adaptive, personal/social, and language). For each area of development, if the developmental age achieved by the child is equal to the actual age, then he/she is considered normal, if exceeds the actual age, he/she is advanced, and if below the actual age, he/she is developmentally delayed. Developmental quotient (DQ) was estimated accordingly [17].

Children were considered blind if visual acuity in both eyes is assessed as worse than 6/60. Children were usually screened for major hearing loss at birth by auditory brain stem response (ABR), and at 7–8 months of corrected age by distraction testing with calibrated noise makers. Those who had not been screened or those with suspected deafness or delayed language at 2 years of age were referred again for audiological assessment.

**Fig. 1:**
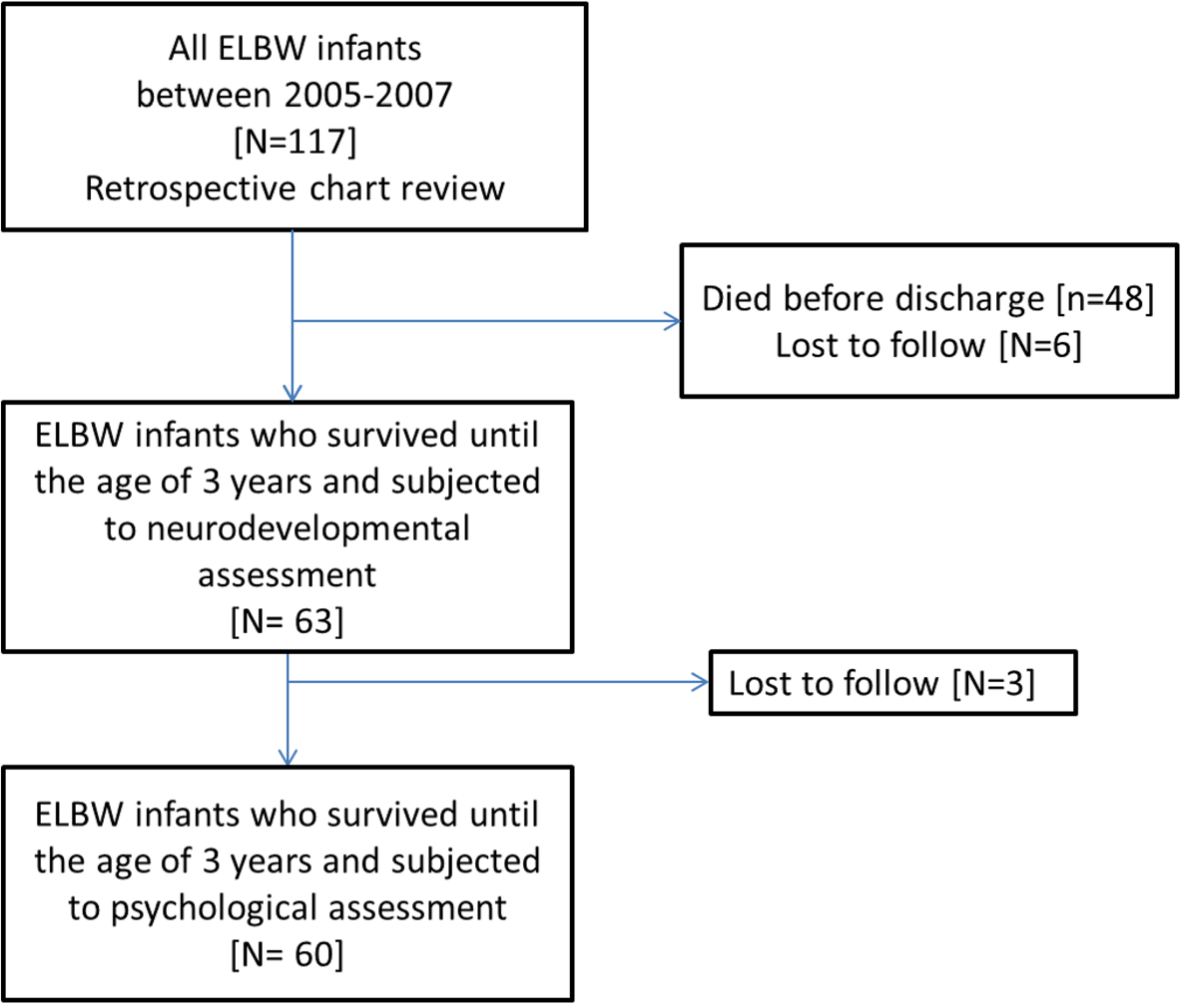
Flowchart of follow up of ELBW infants from birth up to age of 6 years

The psychological assessment includes standardized assessments of cognitive ability, educational progress, and behavior problems. Wechsler Intelligence Scale for Children-Revised (WISC-R) [18] was applied for all ELBW children at the age of six. An Arabic, previously validated version of the scale was used [19]. Children were classified based on the IQ range into: very super (130 and above), super (120–129), high average (110–119), average (90–109), low average (80–89), borderline or slow learner (70–79), mild (55–69), moderate (40–54), and severe intellectual disability (25–39). Figure 1 is a flowchart showing the follow up process of ELBW infants.

The approval of the IRB of the Ministry of National Guard-Health Affairs was obtained to conduct the NICU review (Ref. # RC09/079). Consent to participate was obtained verbally from the parents/guardians of the child participants. Obtaining verbal consent was approved by the IRB.

### Statistical analysis

Demographics and clinical characteristics were summarized and reported for the study cohort. Continuous variables such as; age, weight at birth, GA, etc. were summarized and reported in terms of measures of central tendency (mean and median) as well as measures of variability (i.e. Standard deviation). Categorical variables such as; age gender, etc. were summarized and reported in terms of frequency and proportions. All demographics and clinical characteristics were compared across the study groups using t-test or Chi-square test for continuous and categorical variables, respectively. All results were reported in terms of difference in proportions and means, with corresponding 95% confidence intervals (CI) and *P*-values. All two-way interactions between explanatory variables were tested and found to be non-significant. To test birthweight and GA as predictors of death among ELBW group, a logistic regression model was applied, adjusting for sex, mode of delivery and 5 min Apgar score, and the results were summarized in terms of odds ratios and their corresponding 95% CI. Significance was considered at *p* ≤ 0.05.

## Results

This study was a follow up of 117 ELBW infants for the rate of survival and neurodevelopmental abnormalities. Of all ELBW infants, 69 (59%) survived, and 48 (41%) died, 66 (56.4%) were males, with mean GA of 26.0 ± 1.87 weeks, mean birth weight of 743.60 ± 138.75 g, and mean Apgar score at 5 min of 6.83 ± 2.13. With regard to multiple pregnancies, a total 70.1% of ELBW were the result of singleton pregnancy. Less than one-half of all ELBW (43.6%) were delivered by cesarean section. Diabetes was prevalent in 8.5% of women, and chorioamnionitis in 7.3%. The majority of women were on antenatal steroids (62.7%). Premature rupture of membrane occurred in 20.3% of deliveries. Table 1.

**Table 1:** Early outcomes [death versus survival) in ELBW infants (*n* = 117) according to demographic and biological characteristics

	Dead (*n* = 48, 41%)	Survived (*n* = 69, 59%)	Total (*n* = 117, 100%)	*p*-value
Sex				
male	21(31.8)	45(68.2)	66(56.4)	
female	27(52.9)	24(47.1)	51(43.6)	0.021^@^
Gestational age				
22–23	8(80.0)	2(20.0)	10(8.6)	
24–25	24(55.8)	19(44.2)	43(36.8)	
26–28	12(23.1)	40(76.9)	52(44.4)	
29–30	4(33.3)	8(66.7)	12(10.2)	0.002^@^
Mean ± SD	25.3 ± 2.0	26.4 ± 1.7	26.0 ± 1.9	0.001^b^
Birth weight				
450–600 g.	21(84.0)	4(16.0)	25(21.4)	
601–700 g.	10(47.6)	11(52.4)	21(17.9)	
701–800 g.	8(29.6)	19(70.4)	27(23.1)	
801–995 g.	9(20.5)	35(79.5)	44(37.6)	< 0.001^@^
Mean ± SD	663.0 ± 128.6	799.7 ± 116.8	743.6 ± 138.8	< 0.001^b^
Type of pregnancy				
Single	34(41.5)	48(58.5)	82(70.1)	0.9^@^
Multiple	14(40.0)	21(60.0)	35(29.9)	
Mode of delivery				
CS	15(29.4)	36(70.6)	51(43.6)	
Vaginal.	33(50.0)	33(50.0)	66(56.4)	0.025^@^
Apgar score in 5 min. Mean ± SD	5.7 ± 2.6	7.6 ± 1.3	6.8 ± 2.1	< 0.001^b^
Maternal factors				
Maternal Age (mean, SD)	28.9 ± 7.2	26.6 ± 8.6	28.0 ± 7.2	0.2^b^
Diabetes	5(10.6)	5(7.2)	10 (8.5)	0.5^@^
Premature rupture of membrane	9(18.8)	15(21.7)	24(20.3)	0.7^@^
Chorioamnionitis	5(10.4)	3(4.3)	8 (7.3)	0.2^c^
Prenatal Steroids	29(60.4)	45(65.2)	74 (62.7)	0.6^@^

@---Chi square test, b-----student t test, c-----Fisher exact test

Mortality rate was significantly higher among female infants (*p* = 0.021), those with lowest gestational age (*p* = 0.002), those with lowest birth weight (*p* < 0.001) and those delivered vaginally (*p* = 0.025). With regard to timing of death, one-third died within 3 days after delivery (35.4%) and one-third after 28 days (31.3%). Table 1.

Table 2 shows the distribution of ELBW infants according to neonatal complications. CNS abnormalities were ranked according to their prevalence as follows: IVH (64.4%), Retinopathy of prematurity (44.0%), seizure (21.4%) and encephalopathy (5.1%). CVS anomalies were as follows: PDA (56.9%), indomethacin (17.2%), pulmonary hypertension (10.2%), and CHD (3.4%). Neonatal infections were in form of late onset sepsis (39.8%), nosocomial infections (35.7%), early onset sepsis (9.3%), and meningitis (3.44%). Respiratory morbidities and/or anomalies were as follows: RDS (95.7%), surfactant (87.8%), BPD (36.5%) postnatal steroid (23.3%) and Air leak syndrome (0.9%). The mean IPPV and CPAP were 26.59 ± 22.48 and 16.91 ± 15.45 days respectively. Infant feeding was in the form of formula (6.2%), breast milk (4.0%) or both (64.2%).

**Table 2:** Distribution of ELBW (*n* = 117) according to neonatal complications

Complications	Total (*n* = 117) n, (%)
A-CNS	
IVH	75 (64.1)
PVL	10 (8.5)
Encephalopathy	6 (5.1)
Seizure	25 (21.4)
Retinopathy of prematurity (ROP)	
Grade I	45 (38.8)
Grade II	6 (5.2)
B-CVS	
PDA	66 (56.9)
CHD	4 (3.4)
Indomethacin	20 (17.2)
Pulmonary Hypertension	13 (11.4)
C-Infectious	
Early Onset Sepsis	11 (9.5)
Late Onset Sepsis	47 (40.5)
Nosocomial Infection	41 (35.7)
Meningitis	4 (3.4)
D-Respiratory	
RDS	110 (95.7)
Surfactant	101 (87.8)
Air Leak Syndrom	1 (0.9)
BPD-Bronchopulmonary dysplasia	42 (36.5)
Postnatal Steroid	27 (23.5)
Phototherapy	86 (75.4)
IPPVduration of oxygen exposure (days)	26.6 ± 22.5
CPAPduration of mechanical ventilation (days)	16.9 ± 15.5
Home oxygen	3 (3.5)
E-GIT	
GIT abnormalities	2 (1.8)
Necrotizing enterocolitis (NEC) –stage II	31 (27.0)
Hyperbilirubinemia	82 (71.9)
Phototherapy	86 (75.4)

**Fig. 2:**
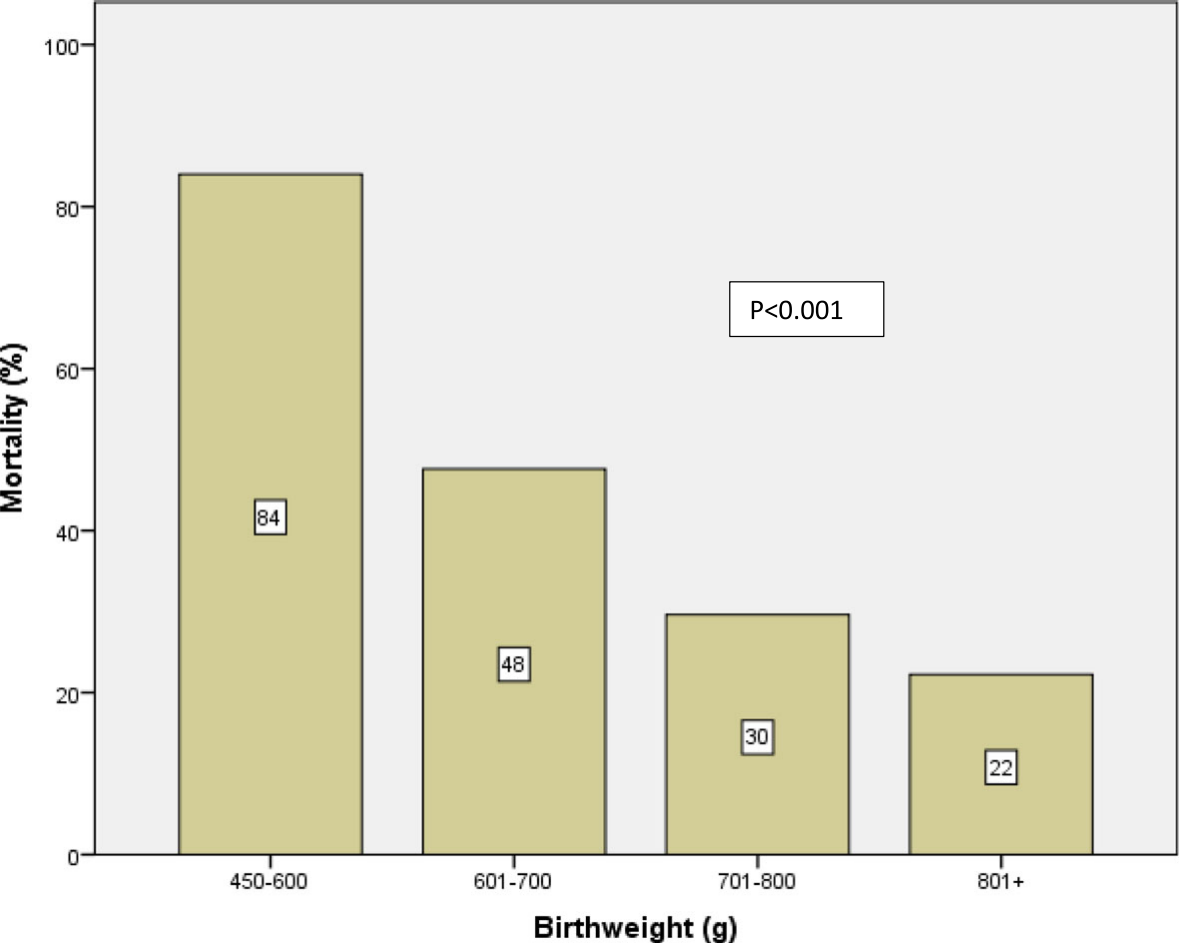
Mortality rate among ELBW infants according to birthweight

Birthweight-specific mortality for all ELBW infants is shown in Fig. 2. Mortality rate decreased steadily with increasing birthweight between 450 and 995 g, ranging from 84% for 450–600 g infants to 22% for 801–995 g infants. The 50% limits of viability was that with a birthweight of > 600 g.

**Fig. 3:**
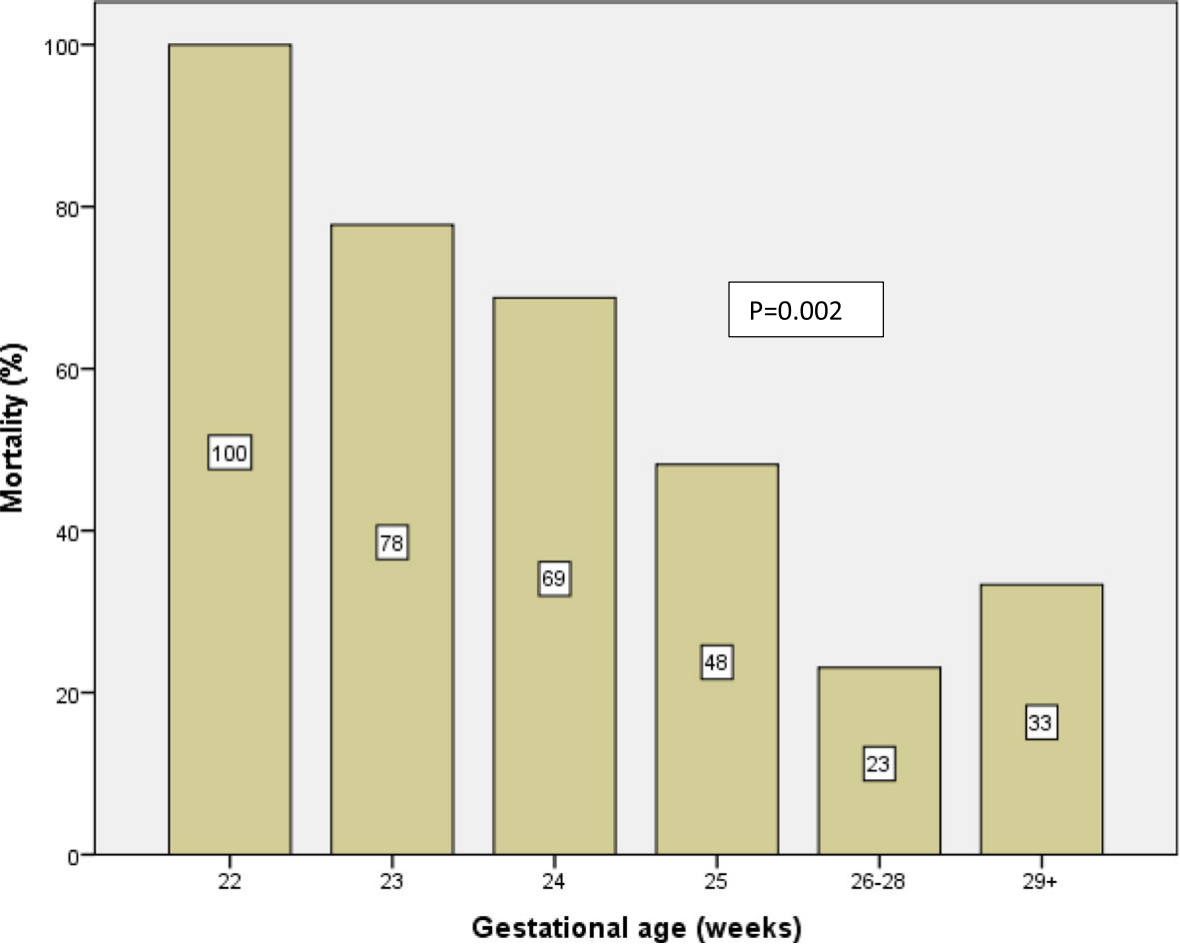
Mortality rate among ELBW infants according to gestational age

The gestational age-specific mortality for all ELBW infants is shown in Fig. 3. Mortality decreased steadily through a gestational age of 28 weeks, ranging from 100% at 22 weeks. to 23% at 26–28 weeks., and then rose slightly for infants between 29 and 30 weeks. The 50% limit of viability was that at week 25 of gestation.

As shown in Table 3, when the association of survival with birthweight and gestational age was investigated in a logistic regression model, adjusting for sex, mode of delivery and 5 min Apgar score, birthweight was a significant predictor of survival (*p* = 0.001), while GA was not (*p* = 0.6).

**Table 3:** Predictors of survival of ELBW infants

Independent variables	*p*-value	Odds ratio	95% C.I.	
			Lower	Upper
Sex	0.1	2.06	.80	5.26
Gestational age	0.6	.914	.65	1.29
Birth weight	0.001*	1.01	1.00	1.01
Mode of delivery	0.9	1.03	.36	2.99
Apgar score in 5 min.	.000*	1.68	1.26	2.25

Table 4 shows the results of pediatric and psychological assessment of the survived ELBW infants. Pediatric assessment by DDST at the age 3 years revealed developmental delays in 38.1% of children [gross motor (14.3%), sensory adaptive (57.1%), personal-social (42.9%) and language (42.9%) domains]. Other impairments were: cerebral palsy (36.2%), delayed speech and stuttering (33.3%), wasting (12.7%), hyperactivity autistic behavior (6.3%) visual disorders (6.3%) and ADHD (3.2%). Psychological assessment using Wechsler test for IQ at the age 6 years revealed that 10% of children were mentally retarded [6.7% mild & 3.3% moderate], in addition to 11.7% slow learners. The mean IQ for ELBW was within the low average range (range, 49–128, mean, 86.75).

**Table 4:** Late outcomes of ELBW children: Results of Pediatric and psychological assessments

	n.	(%)
Normal children	21	34.8
Impairments (at age 3)^a^		
Developmental delay@	24	38.1
Cerebral palsy	23	36.2
Hyperactive autistic	4	6.3
Blindness/amblyopia/amblyopia	4	6.3
Delayed speech/Stuttering	21	33.3
Wasting	8	12.7
ADHD	2	3.2
Psychological assessment (at age 6)^b^		
Superior	3	5.0
Average	42	70.0
Low average	2	3.3
Borderline (Slow learner)	7	11.7
Mild MR	4	6.7
Moderate MR	2	3.3
IQ verbal (mean ± SD)	93.5 ± 21.8	
IQ performance (mean ± SD)	82.8 ± 17.6	
IQ-Full scale (mean ± SD)	86.8 ± 21.3	

a---results for 63 children, b---results for 60 children

@--Developmental delays were: gross motor (14.3%), sensory/adaptive (57.1%), personal/social (42.9%), language (42.9%)

## Discussion

Survival of ELBW neonates has improved with the widespread use of exogenous surfactant agents, maternal steroids, and advancements in neonatal technologies. In our study the overall survival rate was 59%. Mortality rate was associated with both birthweight and gestational age. Mortality rate decreased steadily with increasing birth weight between 450 and 995 g, ranging from 84% for 450–600 g infants to 22% for 801–995 g infants. It decreased steadily through a gestational age of 28 weeks, ranging from 100% at 22 wks. to 23% at 26–28 weeks, and then rose slightly for infants between 29 and 30 weeks, possibly due to the greater incidence of growth retardation and congenital malformations at later gestational ages. The Royal Women’s Hospital in Australia reported death rates of 54% at 23 weeks and 32% at 24 weeks [20]. The corresponding death rate figures in our study were 84% and 48% respectively.

In Japan, where neonatal medical care was the highest quality care in the world, as reported by the WHO in 2005, the viability of ELBW infants with a 50% survival rate, were those at week 23 of gestation or with a birthweight of 500 g [21]. However, in our study, the 50% limits of viability were 25 weeks’ gestation and > 600 g birthweight. It has been summarized in the literature that infants born at < 23 weeks’ gestation and < 500 g are too immature to survive, and provision of care for these infants is unreasonable, while infants who are born at ≥25 weeks’ gestation and with a birth weight of ≥600 g are mature enough and warrant initiation of intensive care [4].

Fetal mortality rates in European countries were higher when based on a 28-week gestational age threshold compared with a 1000 g birthweight threshold [22]. In Japan, the predictors of survival of ELBW infants were GA at delivery, Apgar score at 5 min, antenatal steroid and birthweight [21]. However, in our study, these predictors were birthweight and Apgar score. Fetal growth restriction is a reflection of underlying pregnancy complications [22], and a risk factor of more than 80% of fetal deaths in high-income countries, [23] and may contribute also to lower birthweights [24].

In spite of the reduction in mortality with the use of surfactants, no significant improvement in morbidities such as; chronic lung disease, cerebral palsy, neurosensory deficits and cognitive delays, was reported among surviving infants [9, 25, 26]. In our study, pediatric assessment by DDST revealed high incidence of developmental delays, cerebral palsy, delayed speech and stuttering and wasting. The study by Marlow et al. [7] on 6-year old children born at less than 26 weeks’ gestation in England revealed common cognitive and neurologic impairment with rates ranging from 22 to 34%. The incidence of cerebral palsy among ELBW children in our study is extremely high (36.2%) when compared with figures of 16 to 21% in previous studies [7, 27, 28]. However, direct comparison between our figures and figures of previous studies may be difficult because of the lack of consistency in the outcome measures, and information on the policy of intensive neonatal care and outcome measures in these institutions from which these figures were derived [7, 29, 30].

Psychological assessment using Wechsler test for IQ at the age of 6 years revealed that 10% of children were intellectually disabled [6.7% mild & 3.3% moderate], in addition to 11.7% borderline or slow learners. In Anderson’s study, [30] the mean IQs for ELBW children were in the average range (range, 90–109; mean, 95.5), and they were below the IQs of normal birth weight children. In our study, the mean IQ for ELBW children was below the average range (range, 49–128; mean, 86.75). These findings indicate that adverse cognitive sequelae are a more frequent outcome among such infants, and this would explain the future educational difficulties that have been reported for ELBW children [31].

### Limitations

This study had some limitations and potential biases. First, is that direct comparison between our figures and figures of previous studies may be difficult because of the lack of consistency in operational definitions of the outcome measures, and lack of information on the policy of intensive neonatal care and outcome measures in these studies from which these figures were derived. Second, is that many of the possible factors that influence viability of ELBW infants were not adjusted for in the present study such as; education and adequacy of prenatal care. Maternal anemia, hydramnios/oligohydramnios, incompetent cervix, uterine bleeding, maternal fever, premature rupture of membranes, placental abruption, other excessive bleeding, cephalopelvic disproportion, cord prolapse, and non-reassuring fetal status. Third, is that ELBW children in our study represent the outcomes of an urban tertiary perinatal center and are thus not representative of the Saudi Arabia as a whole. However, our results provide important information for public agencies and health care insurance plans and organizations [32].

## Conclusions

In conclusion, more than one-third of ELBW died before discharge from NICU, and two-thirds of survivors had one or more neurodevelopmental and/or cognitive abnormalities during their first 6 years of life. The study showed that 50% limits of viability of ELBW infants were those at week 25 of gestation or with a birthweight of more than 600 g. These limits are higher than those reported in other studies. However, birth weight, in our study, was the only significant predictor of survival of ELBW infants. These findings might reflect the fact that improving survival rates for the tiniest babies would result in large numbers of disabled children in the community.

Consequently, routine aggressive resuscitation of newborns at 23 weeks and/or with < 600 g. birth weight should be approached with caution. Given the complexity of the issues, the approach to resuscitation of infants at 23 weeks must account for the perspectives of the birth mother and her family, obstetricians, and pediatricians. Thus, the process of care of ELBW needs to be revisited so as to take into consideration the findings of the present study. A multicenter study is recommended to include different types of hospitals in the kingdom and in the region, as the outcome of these infants varies from hospital to another, based on the facilities available and on the attitude of the staff of neonatal intensive care units, whether they are proactive or selective in management of those ELBW infants.

## Abbreviations

ABR: Auditory brain stem response
ADHD: Attention deficit hyperactivity disorder
BPD: Bronchopulmonary dysplasia
CHD: Congenital heart disease
CLD: Chronic lung disease
CNS: Central nervous system
CVS: Cardiovascular system
DDST: Denver developmental screening test
DQ: Developmental quotient
ELBW: Extremely low birth weight
GA: Gestational age
GIT: Gastrointestinal tract
IQ: Intelligent quotient
IRB: Institutional Review Board
IVH: Intraventricular hemorrhage
KAMC: King Abdulaziz Medical city
LSCS: Lower segment cesarean section
MNG-HA: Ministry of National Guard-Health Affairs
MR: Mental retardation
NEC: Necrotizing enterocolitis
NICU: Neonatal intensive care unit
PDA: Patent ductus arteriosus
PVL: Periventricular Leukomalacia
RDS: Respiratory distress syndrome
SD: Standard deviation
SVD: Spontaneous vaginal delivery
WHO: World Health Organization
WISC-R: Wechsler Intelligence Scale for Children-Revised

## References

[1] Lorenz JM (2001). The outcome of extreme prematurity. Semin Perinatol.

[2] Doyle LW (2004). The Victorian infant collaborative study group. Neonatal intensive care at borderline viability. Is it worth it?. E Hum Develop.

[3] Kaiser JR, Tilford JM, Simpson PM, Salhab WA, Rosenfeld CR (2004). Hospital survival of very-low-birth-weight neonates from 1977 to 2000. J Perinatol.

[4] Seri I, Evans J (2008). Limits of viability: definition of the gray zone. J Perinatol.

[5] American Academy of Pediatrics: “Trends in Mortality and Morbidity for Very Low Birth Weight Infants, 1991–1999.” Pediatrics Volume 10, Issue 1 (2002 10.1542/peds.110.1.143Wood NS, 10.1542/peds.110.1.143 12093960

[6] Marlow N, Costeloe K, Gibson AT, Wilkinson AR (2000). Neurologic and developmental disability after extremely preterm birth. EPICure study group. N Engl J Med.

[7] Marlow N, Wolke D, Bracewell MA, Samara M, the EPICure Study Group (2005). Neurologic and developmental disability at six years of age after extremely preterm birth. N Engl J Med.

[8] Marlow N, Wolke D, Bracewell MA, Samara M (2005). Neurologic and developmental disability at six years of age after extremely preterm birth. N Engl J Med.

[9] Hack M, Taylor HG, Drotar D, Schluchter M, Cartar L, Andreias L, Wilson-Costello D, Klein N (2005). Chronic conditions, functional limitations, and special health care needs of school-aged children born with extremely low-birth-weight in the 1990s. JAMA.

[10] Anderson P, Doyle LW, the Victorian Infant Collaborative Study Group (2003). Neurobehavioral Outcomes of School-age Children Born Extremely Low Birth Weight or Very Preterm in the 1990s. JAMA.

[11] Chervenak FA, McCullough LB, Levene MI (2007). An ethically justified, clinically comprehensive approach to peri-viability: gynecological, obstetric, perinatal and neonatal dimensions. J Obstet Gynaecol.

[12] Streiner DL, Saigal S, Burrows E, Stoskopf B, Rosenbaum P (2001). Attitudes of parents and health care professionals toward active treatment of extremely premature infants. Pediatrics.

[13] Prenatal Audit. In: Ed Dunn PM, Mcllwaine G, editors. A report produced for the European association of perinatal medicine, vol. 39: 1996; The Parthenon Publishing Group Ltd, Casterton Hall Carnforth Lancs LA6 2LA United Kingdom. http://www.parthpub.com.

[14] Nishida H (1992). The viability limit of gestation for the fetus and premature neonates. Asian Med J.

[15] Special considerations. In: Braner D, Kattwinkel J, Denson S, Zaichkin J, editors. Textbook of Neonatal Resuscitation. 4th ed. Elk Grove Village, IL: American Heart Association, American Academy of Pediatrics, c2000, Libraries Australia; 2007. p. 7–19.

[16] Al-Alaiyan S, Al-Abdi S, Alallah J, Al-Hazzani F, AlFaleh K (2013). Pre-viable newborns in Saudi Arabia: where are we now and what the future may hold?. Curr Pediatr Rev.

[17] Abolfotouh MA, Nofal LM, Nosseir SA (1990). A simplified method of developmental assessment of infants and preschool children and some associated findings. Alexandria Journal of Pediatrics.

[18] Wolke D, Ratschinski G, Ohrt B, Riegel K (1994). The cognitive outcome of very preterm infants may be poorer than often reported: an empirical investigation of how methodological issues make a big difference. Eur J Pediatr.

[19] Ibrahim ER (ed.). Denver Developmental Screening Test. Manual. Department of Psychology, King Saud University, Riyadh, Saudi Arabia, National Guard Printing Press, 1991.

[20] Batton DG, DeWitte DB, Espinosa R, Swails TL (1998). The impact of fetal compromise on outcome at the border of viability. Am J Obstet Gynecol.

[21] Ogawa M, Matsuda Y, Kanda E, Konno J, Mitani M,Makino Y, Matsui H. Survival Rate of Extremely Low Birth Weight Infants and Its Risk Factors: Case-Control Study in Japan. Obstetrics and Gynecology Volume 2013, Article ID 873563, 6 pages. 10.1155/2013/873563 PMC3858981 24371528

[22] Mohangoo AD, Blondel B, Gissler M, Velebil P, Macfarlane A, Zeitlin J. The Euro-Peristat Scientific Committee. International Comparisons of Fetal and Neonatal Mortality Rates in High-Income Countries: Should Exclusion Thresholds Be Based on Birth Weight or Gestational Age? PLoS One. 2013;8(5) 10.1371/journal.pone.0064869 10.1371/journal.pone.0064869 PMC3658983 23700489

[23] Frøen JF, Gordijn SJ, Abdel-Aleem H, Bergsjø P, Betran A (2009). Making stillbirths count, making numbers talk - issues in data collection for stillbirths. BMC Pregnancy Childbirth.

[24] Chard T (2001). Does the fetus lose weight in utero following fetal death: a study in preterm infants. BJOG.

[25] Anderson P, Doyle LW (2003). Neurobehavioral outcomes of school-age children born extremely low birth weight or very preterm in the 1990s. JAMA.

[26] Subramanian KNS. Extemely Low Birth Weight Infant.” Medscape – Pediatrics: Cardiac Disease and Critical Care Medicine (2014). https://emedicine.medscape.com/article/979717-overview?cc=aHR0cDovL2VtZWRpY2luZS5tZWRzY2FwZS5jb20vYXJ0aWNsZS85Nzk3MTctb3ZlcnZpZXc%3D&cookieCheck=1. Accessed 12 Aug 2018.

[27] Finnstrom O, Gaddlin PO, Leijon I, Samuelsson S, Wadsby M (2003). Very-low-birth-weight children at school age: academic achievement, behavior and self-esteem and relation to risk factors. J Matern Fetal Neonatal Med.

[28] D’Angio CT, Sinkin RA, Stevens TP (2002). Longitudinal, 15-year follow-up of children born at less than 29 weeks’ gestation after introduction of surfactant therapy into a region: neurologic, cognitive, and educational outcomes. Pediatrics.

[29] Hack M, Fanaroff AA (1999). Outcomes of children of extremely low birthweight and gestational age in the 1990’s. Early Hum Dev.

[30] Emsley HC, Wardle SP, Sims DG, Chiswick ML, D’Souza SW (1998). Increased survival and deteriorating developmental outcome in 23 to 25 week old gestation infants, 1990-4 compared with 1984-9. Arch Dis Child Fetal Neonatal Ed.

[31] Saigal S, den Ouden L, Wolke D (2003). School-age outcomes in children who were extremely low birth weight from four international population-based cohorts. Pediatrics.

[32] Martin JA, Hamilton BE, Sutton PD, Ventura SJ, Menacker F, Munson ML (2003). Births: final data for 2002: National Vital Statistics Report 52 no. 10.

